# Embracing and resisting climate identities in the Australian press: Sceptics, scientists and politics

**DOI:** 10.1177/0963662515584287

**Published:** 2015-05-08

**Authors:** Rusi Jaspal, Brigitte Nerlich, Kitty van Vuuren

**Affiliations:** De Montfort University, UK; The University of Nottingham, UK; The University of Queensland, Australia

**Keywords:** climate change, identity, media, scepticism, social representations

## Abstract

This article charts the development of a label that appeared early on in Australian debates on climate change, namely ‘greenhouse sceptics’. We explore who uses the label, for what purposes and with which effects, and how this label may contribute to the development of social representations in the climate debate. Our findings show that over the last 25 years, ‘greenhouse sceptic’ has been used by journalists and climate scientists to negativize those criticizing mainstream climate science, but that it has also been used, even embraced, by Australian climate sceptics to label themselves in order to construct a positive identity modelled on celebrity sceptics in the United States. We found that the label was grounded in religious metaphors that frame mainstream science as a catastrophist and alarmist religious cult. Overall, this article provides detailed insights into the genealogy of climate scepticism in a particular cultural and historical context.

## 1. Introduction

Social scientists (e.g. Jaspal et al., 2013) have studied climate scepticism – doubting aspects of a generally accepted body of climate scientific research - with some examining how scepticism has emerged and developed over time (Weart, 2010), while others focus on labels used in reference to people who accept or doubt the existing science (Howarth and Sharman, 2015). By ‘labels’ we mean words and phrases applied to groups/individuals who take a particular stance on climate change. There has so far been no systematic investigation of how labels have emerged and changed over time and how they may contribute to the development of social representations of climate change.

**Figure 1.**
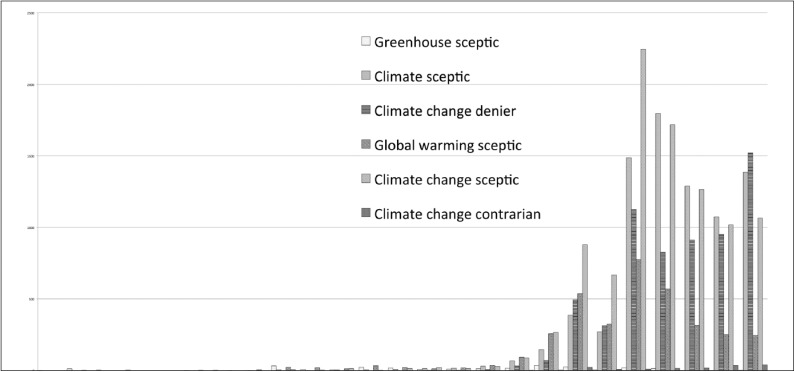
Climate sceptical labels over time; All English Language News; LexisNexis; high similarity setting; consulted on 13 June 2013.

In this article, we investigate how labels are used in relation to ‘climate scepticism’ in the Australian press, focusing on the label ‘greenhouse sceptic’. Surveying the rise and fall of several labels, we found the label ‘greenhouse sceptic’ was among the first used to describe critics of mainstream climate science (Figure 1), and that this label was mainly used in Australia.^1^

The Australian political and media landscape deserves scrutiny, as climate scepticism has rarely been studied in this context. Australia makes one of the highest per capita contributions to emissions, and is vulnerable to climate change impacts (Burgmann and Baer, 2012). Here, we explore how the ‘greenhouse sceptic’ label is used in the press and the potential effects of its use for debates on climate science and politics. Specifically, we ask the following: How did actors writing media articles or quoted in the papers employ this label (to label themselves or others) and thus establish particular types of identities? For what social, political and rhetorical purposes (to make a scientific point, a political point, etc.) are labels employed and how does this shape the climate change debate in Australia? Drawing upon Social Representations Theory, we examine these questions through a thematic analysis of a small corpus of Australian newspaper articles.

### Australia: climate change politics and the media

**Figure 2.**
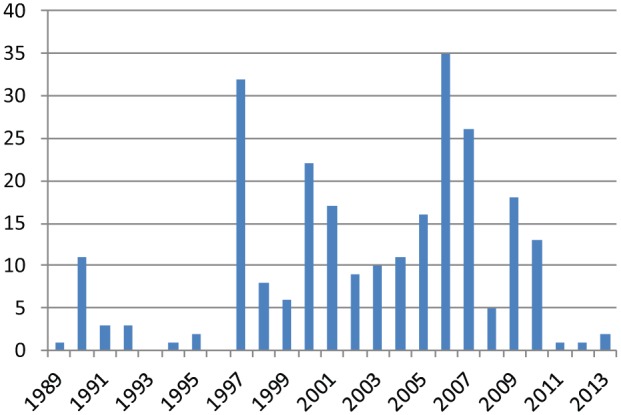
The use of the label ‘greenhouse sceptic’ (‘greenhouse skeptic’) in All English Language News; LexisNexis; high similarity setting; consulted on 13 June 2013.

Climate change has been described as one of the most politicized scientific issues, and this has clearly been the case in Australia. Australia’s two major political parties, the Australian Labor Party and the Liberal Party of Australia, have performed poorly in response to climate change (Talberg, et al., 2013). Following the Australian Academy of Science’s 1976 acknowledgement of anthropogenic climate change, the power of the fossil fuel industries escalated and began to counteract the theory (Burgmann and Baer, 2012). When Australia adopted the 1988 Toronto Target, which aimed for a 20% reduction in greenhouse gas emissions by 2005, legislators included the proviso that reductions should not threaten the economy. The label ‘greenhouse sceptic’ was initially coined in the United States in 1989^2^ but was now used in the Australian press for the first time (Figure 2). The label is a metaphorical compound which can be unpacked as follows: somebody who doubts the increased greenhouse effect leading to global warming. The compound ‘greenhouse effect’ itself stands for ‘enhanced greenhouse effect’. While the earth would be inhabitable without the natural greenhouse effect, the enhanced greenhouse effect might lead to dangerous global warming (Australian Academy of Sciences, 2013). As a metaphor for global warming, the ‘greenhouse effect’ has a history reaching back to the 1800s (Nerlich and Hellsten, 2014).

Under the Keating Labor Government, Australia signed the United Nations Framework Convention on Climate Change (UNFCCC), and Keating established the National Greenhouse Response Strategy (NGRS), including a voluntary scheme to encourage industry to reduce emissions. At Kyoto in 1997, Liberal Environment Minister, Senator Robert Hill, secured a controversial concession which allowed Australia to *increase* its emissions by 8%.^3^ During this debate, we see the second spike in the use of the label. Along with the United States, Liberal Prime Minister Howard refused to ratify the Kyoto Protocol. Compliance with carbon reduction targets was framed in Australian politics as economically detrimental (Kurz et al., 2010). Howard’s governments were closely allied to the ‘carbon lobby’ representing fossil fuel interests who had unparalleled influence over Australian climate policies (Pearse, 2007). His government ‘worked diligently to control public debate’ over the greenhouse effect by denying its existence, sidelining climate research (Hamilton, 2007: 13).

By 2006, when we see the third spike in coverage, climate change was in the top five issues of concern to Australian voters (Pearse, 2007). The previous year had been the hottest on record; a prolonged drought and dwindling water supplies led to water restrictions being imposed on urban populations. In 2006, Al Gore visited Australia to promote his documentary *An Inconvenient Truth*, and the British Government released the Stern Review, both receiving widespread coverage in the Australian media.

Prime Minister Abbott’s electoral success in September 2013, which he labelled as ‘a referendum on the carbon tax’ (Gurney, 2014), brought about further changes in climate change policy. Abbott sought to undermine the climate science underpinning the legislation, by establishing ‘a sense that Australia was on the edge of disaster, carried there by cheats, fakes, crooks and incompetent liars’ (Chubb, 2014: 65). Once elected, Abbott quickly repealed the *Clean Energy Act*, and abolished the Climate Commission supported by the newly elected Palmer United Party.^4^

Australia’s political stance on climate change has been characterized by engagement and disengagement, due to the perceived impact of carbon reduction for the economy, diverging political ideologies and political opportunism. The vicissitudes of Australia’s political engagement are mirrored in print media in debates around climate change (scepticism).

### Climate change (scepticism) in the media

The print media constitute an important source of societal information regarding climate change and can at least set the tone for socio-political engagement with societal issues, by influencing and reflecting policy agendas (Reese et al., 2003). Media-generated controversies may lead to increased scepticism (Boykoff and Boykoff, 2004). Moreover, for researchers, the print media are the only source for achieving a historical understanding of the use of climate sceptic labels before blogs and twitter became commonplace.

Studies have only recently begun to focus on climate scepticism in the mainstream media (Painter and Ashe, 2012). This is in a context in which scientific knowledge about climate change and its causes has increased substantially and in which climate scientists generally agree about what is happening and why, while simultaneously social representations about this issue are increasingly fragmented and polarized.

In a study of Australia’s 10 largest newspapers in 2011/2012, Bacon (2013) found that coverage of climate change in Australia was primarily ‘framed within a vociferous political debate about climate change policy’, with few stories mentioning climate science (p. 10). One-third of articles did not accept the scientific consensus of anthropogenic climate change, and climate scepticism received favourable coverage. However, there is little detailed empirical research into the portrayal of climate change (scepticism) in the Australian media (but see Burgmann and Baer, 2012; Chubb, 2014; Speck, 2010), which, in view of the political complexity of climate change in that country, is an important lacuna.

### ‘Greenhouse sceptic’: label and social representation

In human cognition, labels function as conceptual anchors and stabilize abstract ideas in working memory (Clark, 1997). They simplify perception by allowing us to group people into categories (under labels), and to sort friend from foe, insiders from outsiders and so on (Becker, [1963] 1973). Labels are social – they determine one’s patterns of interaction with the labelled individual/group. For instance, the label ‘denier’ characterizing an individual unconvinced by climate science constructs the individual as knowing but refusing to accept the truth. In addition to other-labelling, people may also label, or categorize, themselves (Chryssochoou, 2003). Self-labels and how we are labelled by others do not always match, which can impede communication and induce intergroup hostility. In our analysis, we are particularly interested in the *social* functions of labelling – that is, how self- and other-labels create particular patterns of meaning, thinking, and interaction vis-à-vis climate change.

Theoretically, we draw upon Social Representations Theory (Moscovici, 1988), which focuses on collective elaborations of knowledge and how cultural meaning systems evolve. A social representation consists of a network of ideas, values and practices in relation to a given social object which can facilitate communication about it. A label may be thought of in terms of a social representation when it is *anchored* to, and *objectified* in terms of, other social phenomena.

- Anchoring refers to the process of making something unfamiliar understandable by linking it to something familiar. For instance, by anchoring climate science to fraud, it is construed as something to be mistrusted.
- Objectification is the process whereby unfamiliar and abstract objects are transformed into concrete and ‘objective’ common-sense realities – most notably through the use of metaphor – allowing us to map aspects of more familiar knowledge (the so-called source domain) onto more unfamiliar knowledge (the so-called target domain) (Nerlich, 2010). ‘The analysis of the relationship between source domain and target domain in a metaphor used as an objectification device can help explain how social representations are acquired collectively and individually’ (Wagner et al., 1995: 671), and, we argue, become part of social/group identities. People not only see the world but also *themselves* through metaphors: use of religious metaphors can serve to construct climate science as speculative and dogmatic, rather than empirically and scientifically grounded. In this case, a pervasive and stable mapping between a source and a target domain, namely, the ‘conceptual metaphor’ Science is Religion (Lakoff and Johnson, 1980), is exploited to frame climate science/scientists in particular ways. This conceptual metaphor is the basis for many expressions, such as ‘the scientific orthodoxy of anthropogenic global warming’, ‘engaging in a witch hunt against global warming heretics’ and so on. Through metaphors we label and comprehend one thing in terms of another; this includes ourselves and our identities.

Labels such as ‘climate sceptic’ or ’scientist’ are not devoid of cultural references. In using particular labels in particular social, political and rhetorical contexts, newspaper articles may anchor climate change in broader cultural constructs, thereby contributing to social representations. Social Representations Theory enables us to emphasize the *social* dimension of labelling by focusing on potential implications of using particular labels in particular ways in particular contexts. Moreover, labels (in the sense of self-categories) can be examined through the lens of the theory given that identity itself arguably constitutes a form of social representation (Chryssochoou, 2003).

We present qualitative analyses of a particular label, and emerging social representations, used in relation to climate change. We provide a longitudinal analysis of three significant temporal points – 1990, 1997 and 2006. Within each year, we begin by outlining the emerging themes relating to the use of the label ‘greenhouse sceptic’ and scrutinize the contexts in which the label is used, for example, whether the label is used positively or negatively, for self-description or other-description, to legitimize or delegitimize individuals/groups.

## 2. Method

### Data collection

Using the news database LexisNexis, we conducted a search of the label ‘greenhouse sceptic’ used in media coverage of climate scepticism in ‘All English Language News’ on a high similarity setting (on 13 July 2013). When originally mapping the use of labels over time, we found that ‘greenhouse sceptic’ was the most popular. We found 252 articles mentioning ‘greenhouse sceptic’, published between 13 October 1989 and 23 April 2013, of which 209 were newspaper articles and the rest newswires, press releases and so on. A total of 158 articles were published in Australian newspapers. There were three spikes in the Australian print media’s use of ‘greenhouse sceptic’, in 1990, 1997 and 2006; we focus on coverage in these seminal years. In total, our corpus included 50 newspaper articles (Table 1).

**Table 1.** Articles included in the three corpora.

Year	Outlet	Number of news items
1990	*The Herald Sun*/*Sunday Herald Sun* (Melbourne)	7
	*The Advertiser*/*Sunday Mail* (Adelaide)	4
	*The Courier Mail*/*Sunday Mail* (Brisbane)	2
1997	*Australian Financial Review* (national)	5
	*The Sydney Morning Herald* (Sydney)	4
	*The Australian* (national)	3
	*The Canberra Times* (Canberra)	2
	*The Courier Mail*/*Sunday Mail* (Brisbane)	1
2006	*The Age* (Melbourne)	7
	*The Australian* (national)	4
	*Australian Financial Review* (national)	4
	*The Canberra Times* (Canberra)	3
	*The Daily Telegraph*/*Sunday Telegraph* (Sydney)	2
	*Hobart Mercury*/*Sunday Tasmanian* (Hobart)	2
	*The Herald Sun* (Melbourne)	1

### Analytic approach

We analysed the corpus using qualitative thematic analysis, ‘a method for identifying, analysing and reporting patterns (themes) within data’ (Braun and Clarke, 2006), represented as themes. Here, thematic analysis is employed to identify emerging social representations of climate scepticism anchored around the term ‘greenhouse sceptic’ (p. 78) and the fine-grained linguistic and rhetorical strategies employed.

We read through the corpus to familiarize ourselves with the broader themes, noting initial observations that captured essential qualities, units of meaning and apparent rhetorical techniques within the corpora. We discussed our respective initial codes, which included inter alia general tone, forms of language (e.g. metaphors), comparisons, categorizations and emerging patterns. Potentially idiosyncratic interpretations of the data were discussed until consensus was reached. Initial codes were collated into preliminary themes. Final themes were arranged into a coherent structure best reflecting the corpora.

## 3. Analysis

We analysed press coverage chronologically in 1990, 1997 and 2006. Here, we chart overall themes relating to science and policy and the use of greenhouse metaphors and religious metaphors in the context of this debate.

### 1990: greenhouse sceptics come in from the cold

In 1990, the first Intergovernmental Panel on Climate Change (IPCC) report was published, which began to generate some debate around climate science and politics. These political events appear not to have had a direct impact on the use of the label ‘greenhouse sceptic’ in the Australian press. Press coverage was instead triggered by a newspaper article published in the prestigious US science magazine *Science* at the end of 1989 amid the publication of a book entitled *The Climate Trap* by an Australian sceptic, John Daly, also in 1989. In 1990, there was a discernible attribution of positive characteristics to the label ‘sceptic’, an emerging delineation of ingroup versus outgroup in the context of climate change and the crystallization of climate identities in relation to representations of alarmism and uncertainty.

#### Imbuing the sceptic label with positive characteristics

An event outside Australia triggered the first use of the label ‘climate sceptic’ in the Australian press.^5^ Malcolm Newell wrote a news item entitled ‘“Hot Air” on greenhouse’, reporting on a *Science* article entitled ‘Greenhouse skeptic out in the cold’.^6^ The *Science* piece focused on one of the most prominent climate sceptics, Richard Lindzen, still active today, referring to him as an ‘iconoclast’ – a rather heroic label repeated several times and amplified in the Australian news article.

Given Lindzen’s personification of the more general category of climate sceptics, his representation as an iconoclast (an individual who destroys images of religious veneration and attacks cherished beliefs as based on fallacy or superstition) rhetorically generalized this religious metaphor to the whole category ‘sceptics’. This metaphor evokes imagery of an individual refusing to conform to fallacy, regardless of its broad acceptance, and who is committed to the revelation of truth. Thus, sceptics were implicitly positioned as heroically pursuing truth in a world dominated by misconceptions (in turn associated with climate scientists). Religious metaphors were employed to construct sceptics as the ‘real’ scientists. This positive use of a religious metaphor to label a climate sceptic in turn serves to frame environmentalists and climate scientists as a religious cult (Nerlich, 2010). Thus, the overarching conceptual metaphor ‘Science is Religion’ can produce many framings of both climate science and scientists, and those who oppose climate science.

Similarly, the *Science* article was quoted as claiming that ‘no other US greenhouse sceptic has such scientific stature’, conferring further legitimacy and gravitas on the label and the group it represents. Although the Australian news article also referred to Lindzen as ‘a doubting Thomas of the “greenhouse” environmental debate’, it attributed to him the identity of a ‘leading American meteorologist’ warning readers not to ‘dismiss him as another crank’. There was a clear delineation of the emerging label climate sceptic and negative categories, stereotypes and representations such as ‘crank’.

In 1990, several other articles stressed the necessity for Australian scientists, politicians and citizens to consider *all* arguments about climate change with care and balance, carving a space for climate sceptics to voice their critique of climate science and in which these critiques can plausibly be accepted (Boykoff and Boykoff, 2004).

In this period of coverage, ‘greenhouse sceptic’ was gradually being constructed in positive terms, laying the groundwork for its deployment as a self-identity category after 1990, that is, as a category that positions the individual in the climate change debate and communicates their position in relation to it (Chryssochoou, 2003).

#### Scientists versus sceptics: power, silencing and prejudice

There was increasing delineation of the sceptics’ ingroup from the climate scientist outgroup. For instance, an article^7^ written by Tom Gosling, a prolific freelance science journalist, uses the label ‘greenhouse sceptic’ in its headline (‘Greenhouse sceptic stirs science congress critics’) in reference, for the first time, to an Australian – John Daly. As pointed out above, in 1989 Daly, a self-taught climate specialist, had published a book entitled *The Greenhouse Trap: Why the Greenhouse Effect Will Not End Life on Earth* (Daly, 1989), in which he identified various aspects of mainstream climate science of which he and others were sceptical. The book referred to ‘the Armageddon Syndrome’, to scientists as ‘False Prophets’, and to ‘the Cult of Experts’.^8^ Consistent with many articles published in this year, the climate scientist outgroup was delegitimized through the use of religious metaphors, which constructed climate science as based on fallacy, ingroup favouritism and even psychopathology.

Controversy arose from the fact that Daly had not been allowed to present a paper at the 59th Australian and New Zealand Association for the Advancement of Science (ANZAAS) conference in 1990, because, as a ‘senior CSIRO scientist said, “He’s not a scientist”’.^9^ Sceptics interpreted this as an act of ‘gagging’,^10^ which constructed climate sceptics as the ‘victims’ of an injustice perpetrated by climate scientists. Use of this metaphor served to represent an undemocratic process of domination led by climate scientists who allegedly sought to forward their own agenda and to silence the voice of the climate sceptic community. Yet, as a newspaper article^11^ highlighted, ‘Mr Daly defended his right to speak at the conference saying he didn’t believe it was necessary to have a PhD or work for an institution to be called a scientist’ – an argument which continues to be deployed by sceptics today (Jaspal et al., 2013). Consistent with the representation of climate sceptics as heroically championing truth over fallacy, here there is a construction of a prominent climate sceptic laying claim to the right to speak openly.

The charge of false prophecy against mainstream climate scientists was reiterated, albeit as a question, in an article^12^ by Gosling entitled ‘Throwing stones at the Greenhouse’:

‘Is the Greenhouse Effect a false prophecy by scientists who have too much faith in their computers. Or is it a sensible warning that something might be going wrong with our planet? According to a new band of “greenhouse dissenters”, it is a false prophesy which carries about as much credibility as tea-leaf reading’ and a ‘load of old codswallop, invented by scientists so they can get research grants’.

Climate science was anchored to religion through the use of the metaphors ‘prophecy’ and ‘faith’ in relation to scientific predictions – the ‘faith’ of scientists was constructed as excessive, the ‘prophecy’ as ‘false’. Although the opposing argument, ‘something might be going wrong with our planet’, was certainly entertained, this was subordinate to the main representation that climate science is religion.

Furthermore, climate science was anchored to less socially accepted and less plausible forms of orthodoxy, namely, speculative superstition, through a comparison with ‘tea-leaf reading’. Climate science was constructed as nonsensical and the scientists’ motive for disseminating ‘codswallop’ as financial greed (Jaspal et al., 2013). Against this backdrop, the ‘greenhouse dissenters’ were positioned as valiantly leading the struggle for truth. In one article,^13^ Gosling referred to Mr Daly as ‘Australia’s most vocal greenhouse skeptic’ and, in another,^14^ as ‘Australia’s leading greenhouse sceptic’. In short, the label ‘sceptic’ was attributed to others, rather than as a self-label, but was increasingly framed in positive terms.

In an outspokenly sceptical article^15^ entitled ‘Global warmth leaves me cold’, D. Hampson referred to Daly as a ‘marine electronics engineer [who] has studied global atmospherics for 20 years’, accentuating his long-standing expertise and justifying Daly’s claims to scientific credibility. In view of this positive construal of a climate sceptic, his exclusion from ANZAAS was represented as unreasonable:

Now why should all these well known and no doubt well funded ‘scientists’ want a lone Greenhouse sceptic gagged? They couldn’t have something to hide, could they? It couldn’t be their own selves, could it, who have been misinforming the community on a massive scale.

This extract exemplifies the construction of a particular pattern of power relations between climate scientists and climate sceptics. While the climate scientists were represented as ‘well known’ and ‘well funded’ and, thus, powerful, climate sceptics were depicted as ‘lone’ actors who were oppressed and, thus, relatively powerless. The label was employed to create a positive identity for greenhouse sceptics, as it evoked imagery of the rather heroic ‘lone ranger’ battling greed and corruption. Greenhouse sceptics like Daly were portrayed as revealing evidence that mainstream scientists try to suppress or keep secret – a trope that would become a mainstay of sceptic discourse after ‘climategate’ in 2009 (Nerlich, 2010). Mainstream scientists, by contrast, were portrayed as being involved in a ‘scam’, a word that rapidly rose to prominence around that period (Nerlich and Koteyko, 2009). Social representations are invoked in order to legitimize the emerging ingroup identity and to delegitimize the outgroup.

#### Emerging representations and identities: alarmism and uncertainty

The emerging climate sceptic identity was based on two major premises, namely, (1) the argument against what in 1990 is increasingly referred to as alarmism and (2) the emphasis upon uncertainty in science in an attempt to delegitimize the ‘alarmist’ predictions of climate science. Thus, the primary ethos of the climate sceptic identity was to legitimize the sceptics’ own *polemic* social representations of climate change and to delegitimize hegemonic social representations of climate change, disseminated by climate scientists.

The social representation of mainstream climate science as alarmist, through its anchoring to doomsday phenomena, came strongly to the fore in 1990, exemplified by the article focusing upon Lindzen’s portrayal in *Science*.^16^ The article reported that Lindzen’s doubts regarding the greenhouse effect were based on critiquing the ‘doomsday scenario’, ‘doomsday predictions’ and the ‘doomsday view’ promulgated by climate scientists. Lindzen was also quoted as stressing the many uncertainties surrounding such ‘predictions’. Thus, like other ‘lone greenhouse sceptics’, he was represented as contesting allegedly exaggerated, fear-mongering predictions. Consistent with the observed focus on religious metaphors in this period, the characterization of these predictions as doomsday evoked religious imagery.

Conversely, the climate science identity emerged from the reinforcement of the hegemonic social representation of anthropogenic climate change. An article^17^ by Gosling quite explicitly argued against the sceptics’ stance,

the scientists have taken the morally responsible line in informing the community of what they believe could be an impending disaster. It is far worse to deny the existence of a problem than to perhaps exaggerate it. We have been warned.

In the extract, the identity of climate scientists was hinged on morality and responsibility – positive values (Irwin, 1995). Their principal aim was represented as averting what ‘could be an impending disaster’, rather than greed as hypothesized by some climate sceptics. Therefore, the climate scientist identity was constructed in opposition to the polemic social representations disseminated by climate sceptics. What was deemed as alarmism by the sceptics was re-constructed as a warning based on morality and responsibility. There is, thus, a competing not only of identities but also of social representations associated with them.

The *Science*/sceptic episode is called metaphorically, ‘a volley in the greenhouse wars’.^18^ This metaphor of war would run through the next few years during which mainstream scientists and greenhouse sceptics struggle over public perception and policy impact and over the positive or negative use of the label ‘greenhouse sceptic’.

An article about Daly^19^ referred to a communication dilemma emerging from a clash between mainstream scientists’ and sceptics’ respective social representations. Gosling quoted the president of the ANZAAS congress,

‘if it turns out in 10 years that the [greenhouse] effect is not severe, scientists will probably be dismissed as a “bunch of wankers”. But if we said nothing, and it turns out that there is very serious global warming, the community will have lost 10 years valuable lead time, and we’ll be condemned as being typical reticent scientists. We’re damned if we do, and dammed if we don’t.’^20^

There was an attempt to safeguard the position of scientists, who faced public scrutiny over the uncertainties underpinning climate science, by constructing the ingroup as being in a no win situation. An element of victimhood was attributed to the ingroup versus that of the sceptics’ outgroup, which is interesting considering the victimhood attributed to the sceptic community described above.

Uncertainty was a pervasive rhetorical tool used in the greenhouse debate of that time (Antilla, 2005), but scientists tried to rhetorically decrease its social and political clout in delegitimizing climate skepticism.

### 1997: greenhouse scepticism gains momentum in Australia

In 1997, the Kyoto protocol was heavily debated, with the United States under Bill Clinton and Al Gore promoting it, and Australia under Liberal Prime Minister John Howard sidestepping it. In this section, we outline the role of Gelbspan’s (1997) book *The Heat Is On* in the framing of scepticism; how the sceptic label became a positive self-identity label; and the crystallization of conflict between climate science and climate sceptic identities, respectively.

#### Gelbspan and the framing of greenhouse sceptics

In early 1997, there was some negative coverage of greenhouse sceptics based on Gelbspan’s book. One article^21^ claimed,

Longtime Boston Globe editor Ross Gelbspan has been riding high since April when Addison-Wesley published his book The Heat is on: The high stakes battle over earth’s threatened climate. Gelbspan’s publisher claims that oil and coal interests have been ‘paying off scientists to pose as “greenhouse sceptics”, employing political lobbyists, proliferating propaganda materials, and distorting and suppressing the mounting evidence of impending climatic disaster’.

While Gelbspan tried to undermine the reputation of greenhouse sceptics in the United States in 1997 by revealing financial ties to fossil fuel industries, Australian greenhouse sceptics began to ally themselves more with their US counterparts that same year, especially during two conferences. In this context, the Australian press criticized Gelbspan’s stance as ‘greenhouse bullying’.

One conference, ‘widely criticised as a partisan showcase for greenhouse sceptics’, was held at Monash University in the autumn of 1997 under the title ‘Greenhouse ’87: Planning for climate change’.^22^ The article reporting on this conference rightly pointed out, ‘All is beautifully poised for an “end of millennium” conflict which will reaffirm the cultural differences between science, which talks in terms of levels of uncertainty, and politics, which craves the illusion of certainty’. There was a sense at the end of 1997 that, as the headline of this article by Leigh Dalton states, ‘Science’s climate of doubt is over’ and that the ‘greenhouse showdown’ had been won by the scientists rather than the sceptics.^23^ Moreover, uncertainty was acknowledged as an inherent aspect of science and, thus, the sceptics’ social representation that climate science is consequently faulty was challenged. It was also acknowledged that the ‘doom-gloom scenarios for which they [climate scientists] are pilloried could be far worse’.^24^

Social representations of climate scepticism were rendered largely problematic in the Australian debate. However, self-alignment with US sceptics provided scope for protecting and enhancing the sceptic identity in a context of growing opposition.

#### Deriving pride from the greenhouse sceptic label

In 1997, the greenhouse sceptic label was beginning to be employed as a self-label. This was observable in coverage of the *Countdown to Kyoto* conference, which became a flashpoint for debate. The conference was organized by a ‘the United States lobby group, the *Frontiers of Freedom*, and the APEC [Australian Asia Pacific Economic Cooperation] Study at Monash University’ in August 1997.^25^ According to Beder,^26^ the ‘conference […] was organised […] to “bolster support” for the Government’s increasingly isolated position on global warming in preparation for the Kyoto conference’. In a related article, US Senator Wallop, Chair of the conference, was labelled a ‘self-confessed greenhouse sceptic’.^27^ This signalled a shift towards using the label greenhouse sceptic positively as an honorific to forge a more assertive sceptical identity.

The main issue of contention was the attendance at this conference of a ‘leading [U.S.] greenhouse sceptic, US congressman John Dingell’.^28^ An article by Ian McPhedran,^29^ entitled ‘Ministers join Greenhouse sceptics at conference’, reported that two ‘senior federal ministers will share the stage with a number of strident anti-environmental movement advocates’. Another article^30^ referred to these critics as defying ‘greenhouse bullying’ by mainstream scientists. In an article entitled ‘Counter Culture’,^31^ Fred Pearce used the label ‘greenhouse sceptic’ positively, portraying the sceptic Patrick Michaels, from US think tank the Cato Institute, ‘a belligerent sceptic’, as a ‘sceptic guru’ and as being in ‘ebullient mood’. Pearce wrote that greenhouse sceptics ‘don’t believe in global warming. And they think their time has come’.

In these extracts, the category ‘greenhouse sceptic’ was more overtly imbued with positive characteristics than in media reporting in 1990 – there was, for instance, reference to a leading sceptic, suggesting a hierarchy within this honorific category. Sceptics were constructed as having ascended the hierarchy of climate change commentators through their association with government ministers, ‘senior federal ministers’. More generally, the increasingly positive category ‘sceptic’ was represented as a valiant one whose members refused to be undermined by ‘bully’ climate scientists.

Ingroup–outgroup dynamics were reiterated, on one hand, and there was a positivization of the climate sceptic identity, increasingly endorsed by sceptics themselves, on the other hand. The sceptic identity is an empowering one, as members believe that they are winning the battle for truth. This episode also shows that Australian policy makers were being influenced by, and integrated into, an emerging Australian/US greenhouse sceptical movement.

### 2006: Australian greenhouse sceptics and the emergence of a counter-movement

The year 2006 was the year of the Fourth IPCC report, the Stern report and several other reports and initiatives. By this point, the social representation of anthropogenic climate change was established as a hegemonic one, discernibly in competition with the polemic representations of climate sceptics. Religious metaphors again emerged this time in the framing and labelling of greenhouse sceptics. In a context where mainstream science was widely seen as winning the war, Rupert Murdoch, then Chairman and CEO of News Corporation, was portrayed as having ‘seen the light’,^32^ as having been ‘converted’ to mainstream climate science and policy,^33^ while the then Federal Environment Minister Ian Campbell was called ‘a born-again believer in the greenhouse crisis’ and Prime Minister Howard ‘among the last of the so-called “greenhouse sceptics” to show signs of recanting’.^34^

Ian Macfarlane, then Industry Minister, a ‘self-described greenhouse sceptic’ was said to have been seeing the positive side of nuclear power, and Victorian State Liberal premier Ted Baillieu was reported to have rejected the claim that he is a ‘greenhouse sceptic’.^35^ There was even talk of a ‘dwindling band of greenhouse sceptics’.^36^ Prominent greenhouse sceptic and editor-in-chief of *The Australian*, Chris Mitchell, was now referred to as ‘one of the “dirty dozens” of Australia’s “greenhouse sceptics”’.^37^ While in 1997, greenhouse sceptics appeared to be making their mark on Australian climate politics, by 2006, the identity ‘sceptic’ was increasingly rejected as a self-label.

In 2006, a discussion began about the labels used by critics of both sides in reference to themselves and others. Some mainstream scientists, such as geologist Chris Sharples, began to question the label ‘sceptic’:

I prefer not to call them sceptics because sceptics demand evidence before coming to a conclusion […] I call them contrarians because they ignore the evidence and just don’t want to know about it.^38^

There was contestation of the self-label ‘sceptic’ employed for self-definition among those who reject mainstream climate science.^39^ By 1997, the self-label had acquired more favourable connotations, but was rejected by scientists such as Sharples who wanted to reserve that label for mainstream scientists who, implicitly, are framed as the real ‘sceptics’. Sharples distinguished between scepticism about scientific evidence and ‘ignorance’ of the evidence and uncritical rejection, and positioned those who view themselves as sceptics within the latter camp, constituting a delegitimization strategy (Bar-Tal, 1990).

There was a backlash by sceptics against a new type of labelling (as ‘deniers’). For instance, Christopher Pearson wrote an article entitled ‘Rising tide of bad science’,^40^ in which he argued against the ‘true believers in catastrophist science’ (again drawing upon religious metaphors) and the ‘catastrophist dogma’ (echoing imagery of alarmism). In particular, he upbraided a columnist working for *The Sydney Morning Herald*, who had written, ‘At least the vocal deniers are shrinking like the Wicked Witch of the West, drenched by a bucket of melting icecaps’, which resisted the ‘sceptic’ label and instead constructed those who reject the hegemonic social representation of climate change as pathologically deflecting truth in favour of fantasy. In response, Pearson wrote,

… Greenhouse gas sceptics, have, at the stroke of a pen, been turned into deniers, the moral equivalent of anti-Semites, along with David Irving and the pseudo-historians who say the Holocaust never happened.

The sceptic identity had, over several years, transformed from an other-label to a self-label imbued with positive characteristics and worthy of pride. Like Pearson, self-identified sceptics therefore took offence at others disputing the label. The ‘denier’ label is anchored to antisemitism and Holocaust denial, which serves as a legitimate rationale for rejecting it and for denigrating those who attempt to attribute the label to sceptics (Bar-Tal, 1990). Yet, despite protests by sceptics, the label ‘climate denier’ is in the process of outperforming almost all other labels apart from climate sceptic (Figure 1).

Although in 2006 there was a sense that mainstream climate science might be winning the argument about climate change, there were also signs of an emerging counter-movement, led by Ian Plimer, ‘head of earth and environmental sciences at the University of Adelaide’. Plimer was quoted in the article just mentioned as ‘an eminent greenhouse sceptic with a nice turn of phrase who’s had plenty to say on this subject in the *Independent Weekly* and deserves a wider audience’. Here, the phrase ‘eminent greenhouse sceptic’ was presented as a positive label with which one could identify and be identified by others – an identity that is embraced, rather than resisted. As demonstrated in this article, this has been facilitated through its anchoring to positive phenomena and its objectification using more positive metaphors.

## 4. Discussion

Our study of the ‘greenhouse sceptic’ label in the Australian press over the past 25 years has shown that climate scientists and those critical of the scientific consensus use the label in a discourse that draws heavily on religious metaphors. The label has been used primarily in an Australian context, though influenced by similar debates in the United States. This article charts the emergence, development and crystallization of the greenhouse sceptic label and demonstrates the label’s social, cultural and rhetorical vicissitudes in the Australia media debate. In recent years, the label has transformed into a *social representation* which evokes cultural, historical and social images (Moscovici, 1988).

### Greenhouse sceptic: emergence of a label

Between 1990 and 2006, the label ‘greenhouse sceptic’ for those sceptical of climate science and policies became prominent in Australian media. In media reporting, it has performed two distinct functions: (1) to label those seen to be outside the perceived consensus about climate change, or (2) to label those fighting this consensus and exposing its perceived flaws, especially in relation to uncertainty and alarmism. Through a variety of rhetorical strategies, including use of religious metaphors and attribution of positive traits to the sceptic label, the groundwork was laid for the construction of a positive sceptic identity.

In 1990, there was an emerging struggle between the hegemonic social representation of anthropogenic climate change and the competing polemic representation of climate scepticism. Scientists and sceptics were constructed as two distinct social groups – as individuals who perceive a bond with like-minded others in their pursuit of a common goal (Johnson and Johnson, 1987) – with distinct, competing social representations organized primarily around truth and fallacy. There was a focus upon the themes of alarmism and uncertainty, which served to delegitimize climate science and to *legitimize* climate scepticism (Bar-Tal, 1990); climate scientists were delegitimized as alarmists and were equated with environmentalists, which put them at odds with dominant political interests.

In the 1997 corpus, greenhouse scepticism gained momentum in the Australian press through two principal paths. First, the book by Gelbspan, intended to delegitimize social representations of climate scepticism, was in fact employed as a stimulus for *asserting* sceptical representations, and climate scientists were branded bullies. Moreover, the label ‘greenhouse sceptic’ came to be used with pride, despite the fact that the label was used negatively in the book sparking this debate.

In this coverage period, the sceptic label became one for self-definition given that for several years a context had been created for self-identification as a sceptic. There was a shift towards the use of ‘greenhouse sceptic’ as an honorific: an empowering self-label. ‘Greenhouse sceptics’ constructed their association with government ministers as evidence of having ascended the hierarchy in the climate change debate. They were now legitimate competitors in the climate change debate and had further pushed climate scientists – depicted as ‘greenhouse bullies’ – towards the periphery of this debate. In addition to becoming a label that differentiates ‘us’ from ‘them’, the sceptic label also acquired the potential to provide self-efficacy and self-esteem in a social, political and rhetorical context in which these very principles of identity had been challenged due, for instance, to perceived ingroup delegitimization (Breakwell, 1986). Articles published during this period and, particularly, those published in response to Gelbspan’s seminal book accentuated the divide between ingroup and outgroup, and illustrated a more assertive sceptical identity.

By 2006, climate change had become a particularly important topic in the Australian press, and some Australian commentators adopted the sceptic label to mount a counter-movement to mainstream science and especially Australian policies based on it. Although the label was no longer used so prominently in Australia, it contributed, we argue, to catalyzing an Australian sceptical identity. The label itself contributed to the broader social representation of scepticism, which may no longer be viewed as a mere polemic representation in the Australian context but rather as having gained momentum and political importance. Moreover, the sceptic identity was clearly being constructed *in opposition to* climate scientists and those who accept mainstream science. The sceptic label now evoked imagery of defiance and distinctiveness vis-à-vis climate scientists and was increasingly being embraced as a self-label.

By 2006, several political developments provided a context for greater debate and defence of social representations of climate change, thereby reigniting the importance of the sceptic label. It was Prime Minister Howard’s final year in office, amid important climate-related world events, such as the IPCC’s publication of its latest climate report, the release of the Stern report in Britain and Al Gore’s film *The Inconvenient Truth*, combined with Australia experiencing its worst drought on record. Climate scientists attempted to re-appropriate the ‘sceptic’ label and to re-label ‘greenhouse sceptics’ as ‘contrarians’ and ‘deniers’ in order to contest their social representations.

### Social representation, metaphor and policy

The use of religious labels and metaphors in the construction of climate identities performs important rhetorical functions. Use of such metaphors serves to delegitimize climate science and to separate out ‘good’ scientists from ‘bad’ scientists, given the presence of scientists in the sceptics camp, but in the process it runs the risk of delegitimizing science itself (Irwin, 1995). This is especially important in the policy sphere, where politicians and administrators may rely on science for making ‘good’ policy and decisions.

The articles appear to frame science as ideology, and vested interest, concepts easily understood by journalists and audiences. By aligning particular sciences with particular political interests, delegitimization can enhance electoral success. In this battle to disseminate their respective social representations to the wider public and political spheres, the two social groups – mainstream climate scientists and climate sceptics – exploit two social representations: science as uncertain, unsettled and alarmist, on one hand, and science being based on an emerging consensus as well as caution, on the other. Sceptics employ conferences as tools to distribute such representations, while mainstream scientists write or refer to books that expose the industrial funding of such conferences and the sceptics who attend them. While sceptics adopt the label ‘greenhouse sceptic’ for self-definition (‘self-confessed’) and invoke their fight against ‘greenhouse bullying’, mainstream scientists label them as ‘belligerent’ or ‘strident’, minimizing the threat they pose by calling them a ‘handful’.

On both sides of the debate, there are clear rhetorical attempts to delegitimize outgroups and to legitimize the ingroup, with a view to resisting the outgroup’s social representations and enhancing the credibility of one’s own. A principal means of doing so among climate sceptics is to anchor climate science to uncertainty, politics, fraud and greed, while an emerging delegitimization strategy among mainstream climate scientists constitutes the anchoring of scepticism to denial. Individuals require a sense of identity validation from others, and contestation from outgroups, in particular, can challenge the self-concept (Breakwell, 1986). Others’ attribution of a label with which one does not identify can induce threats to identity and widen the communicative gap between social groups, thereby accentuating intergroup conflict and polarization (Tajfel and Turner, 1979).

The overarching aim of this study was to explore the use and development of the label ‘greenhouse sceptic’ and its potential implications for social representations of climate change. The print media make an important contribution to social representations because, collectively, they constitute ‘a forum for the discourses of others and [are] a speaker in their own right’ (Carvalho, 2007: 224). They provide space for notions, images and theories to become popularized in the cultures and minds of individuals. The media reports analysed demonstrate powerfully that over time the climate sceptic identity has become a social representation in its own right, providing ‘common sense knowledge about the self’ and indeed about others (Chryssochoou, 2003: 227). This identity/representation imbues the self with esteem, distinctiveness from others and efficacy (Breakwell, 1986). Indeed, having laid the social foundations for a positive climate sceptic identity, sceptics protected and enhanced the label in media reporting and the term ‘denier’, for instance, was rejected. Yet, social representations (and indeed identities) are also co-constructed, contested or supported through other social media – political rhetoric, literature, education, everyday talk and so on. There is a dynamic interplay between media representation, identity and personal experience (Smith and Joffe, 2013). Consequently, the data presented in this article exhibit only one facet of the story regarding social representations of climate change and its actors, but we argue that this is an important facet – one that can perform an agenda-setting function and introduce representations that are later taken up in social and political debates about climate change in online and offline settings (Olausson, 2011). Future research should examine the deployment and development of the ‘greenhouse sceptic’ label in other forms of discourse, such as the first-hand accounts of sceptics themselves and those of individuals who accept the theory of anthropogenic climate change.

This article shows how labels and the metaphors that surround them become social representations and play an important role in the climate change debate, as they separate actors into sides and determine the credibility with which their contributions to the debate on climate change ought to be viewed. Labels can be used to construct identities and to imbue these identities with meaning and value, but they can also inhibit debate and lead to political paralysis – leaving both sides at loggerheads, rather than willing to talk.

## Notes

1. Fluctuations in labels overall will be explored in a different study.

2. *The Associated Press*, 1989.

3.
http://www.smh.com.au/federal-politics/political-opinion/the-australia-clause-bites-back-20091023-hda9.html

4.
http://www.environment.gov.au/climate-change/emissions-reduction-fund

5. *The Advertiser*, 13 January 1990.

6. *Science*, 1 December 1989.

7. *The Herald*, 14 February 1990.

8.
https://quadrant.org.au/opinion/doomed-planet/2009/11/john-l-daly/

9. *The Herald*, 14 February 1990.

10. *Sunday Herald*, 25 February 1990.

11. *The Herald*, 14 February 1990.

12. *The Advertiser*, 10 March 1990.

13. *The Herald*, 21 February 1990.

14. *The Herald*, 28 March 1990.

15. *The Sunday Herald*, 25 February 1990.

16. *The Advertiser*, 13 January 1990.

17. *Herald*, 21 February 1990.

18. *The Advertiser*, 13 January 1990.

19. *The Herald*, 14 February 1990.

20. *Herald*, 14 February 1990.

21. *The Australian*, 20 August 1997.

22. *Canberra Times*, 18 October 1997.

23. *Sydney Morning Herald*, 24 November 1997.

24. *Sydney Morning Herald*, 24 November 1997.

25. *Canberra Times*, 18 August 1997.

26.
http://www.uow.edu.au/~sharonb/STS218/greenhouse/conferences.html

27. *The Australian*, 20 August 1997.

28. *Australian Financial Review*, 30 June 1997.

29. *Canberra Times*, 18 August 1997.

30. *The Australian*, 20 August 1997.

31. *Courier Mail*, 20 September 1997.

32. *Australian Financial Review*, 22 September 2006.

33. *The Age*, 23 October 2006; *Australian Financial Review*, 7 November 2006.

34. *The Age*, 7 October 2006.

35. *The Age*, 25 October 2006.

36. *Canberra Times*, 12 September 2006.

37. *The Australian*, 21 February 2006.

38. *Sunday Tasmanian*, 23 July 2006.

39.
http://www.yaleclimateconnections.org/2012/08/denier-alarmist-warmist-contrarian-confusionist-believer/

40. *Weekend Australian*, 10 June 2006.
